# Fostering and sustaining transnational solidarities for transformative social change: Advancing community psychology research and action

**DOI:** 10.1002/ajcp.12602

**Published:** 2022-06-16

**Authors:** Christopher C. Sonn, Rachael Fox, Samuel Keast, Mohi Rua

**Affiliations:** ^1^ Institute of Health and Sport Victoria University Melbourne Australia; ^2^ School of Psychology Charles Sturt University Wagga Australia; ^3^ Maori and Psychology Research Unit University of Waikato Hamilton Aotearoa New Zealand

**Keywords:** decolonial, radical imagination, re‐empower, solidarities, transnational

## Abstract

As we planned this special issue, the world was in the midst of a pandemic, one which brought into sharp focus many of the pre‐existing economic, social, and climate crises, as well as, trends of widening economic and social inequalities. The pandemic also brought to the forefront an epistemic crisis that continues to decentre certain knowledges while maintaining the hegemony of Eurocentric ways of knowing and being. Thus, we set out to explore the possibilities that come with widening our ecology of knowledge and approaches to inquiry, including the power of critical reflective praxis and consciousness, and the important practices of repowering marginalised and oppressed groups. In this paper, we highlight scholarship that reflects a breadth of theories, methods, and practices that forge alliances, in and outside the academy, in different solidarity relationships toward liberation and wellbeing. Our desire as co‐editors was not to endorse the plurality of solidarities expressed in the papers as an unyielding methodological or conceptual framework, but rather to hold them lightly within thematic spaces as invitations for readers to consider. Through editorial collaboration, we arrived at the following three thematic spaces: (1) ecologies of being and knowledge: Indigenous knowledge, networks, and plurilogues; (2) naming coloniality in context: Histories in the present and a wide lens; (3) relational knowledge practices: Creative joy of knowing beyond disciplines. From these thematic spaces we conclude that through repowering epistemic communities and narratives rooted in truth‐telling, a plurality of solidarities are fostered and sustained locally and transnationally. Underpinned by an ethic of care, solidarity relationships are simultaneously unsettling dominant forms of knowledge and embrace ways of knowing and being that advances dignity, community, and nonviolence.

## INTRODUCTION

Our vision for this issue was to bring into dialogue scholarship, activism, and critical praxis from various corners of the world in mutual exchange. We aimed to advance our field by celebrating the highly relevant contributions to activism, scholarship, and critical inquiry, while simultaneously unsettling activism, scholarship, and critical inquiry as a pathway to empowerment, communality, and healing. With this in mind, we set out to explore the possibilities that come with widening our ecology of knowledge and approaches, the power of critical reflective praxis and consciousness, and the important practices of resistance and re‐existence in contexts where structures of power continue to diminish the ways of being for minoritized, objectified, and racialized cultural groups. Re‐existence is a decolonial concept that includes how people resist oppression, but also goes beyond this to show resurgent practices aimed at the “redefining and re‐signifying of life in conditions of dignity in self‐determination” (Alban in Mignolo & Walsh, 2018, p. 18). Here the notion of re‐empower is useful, where indigenous knowledge systems are prioritized to renarrate colonial spaces in a process toward self‐determination and transformative praxis (Smith, 2017). In this issue, we sought to capture some of the breadth of theories, methods, practices, and their interactions in how those in and outside the academy come together in alliances, in solidarity, to do the difficult work needed to foster liberatory practices. Through this, we seek to live and work without and against systems of violence, while not enacting such systems of violence on others.

Critical scholars and activists from various contexts have pointed to the role of knowledge production in colonizing practices and processes and the need to reclaim and retrieve ways of knowing, doing, and being from regions of the world that have been referred to as the Global South within the context of a world system (e.g., Mignolo & Walsh, 2018; Smith, 2012). For many scholars and activists, this critical work entails making visible and transforming the hegemonic Western Eurocentric approaches to theory, research and practice, and centering and critically engaging with the diversity of paradigms and approaches that have been neglected, othered or erased. In the literature across fields, authors have highlighted the diverse roots of counterhegemonic scholarship. Such scholarship informs the current resurgent decolonial moment that has been described as a paradigm shift that has the potential to disrupt colonial legacies of power, knowledge, and being (Mignolo & Walsh, 2018). This is a multidimensional project—to critique and challenge the suffocating and brutal hegemonies of colonial systems of knowledge and life, to lift up and animate the smoldering yet vibrant embers of knowledge still alive in communities under siege, and to build praxis across the place, to ask what else is possible. There are rich, yet often ignored and omitted knowledge archives, including what Raewyn Connell (2007) referred to as *Southern Theory*, that are critical of these absences, and that offer alternative worldviews and orientations to those rooted in structures of modernity, capitalism, and patriarchy. Writing with reference to research and indigenous peoples, Linda Tuhiwai Smith (2012) noted that decolonization “has not meant a total rejection of all theory or research or Western knowledge. Rather, it is about centering our concerns and world views and then coming to know and understand theory and research from our own perspectives and for our own purposes” (p. 41).

At the Seventh International Conference of Community Psychology (ICCP) held in Santiago, Chile, in 2018 and the 2019 Society for Community Research and Action (SCRA) Biennial Conference held in Chicago, United States, there were strong calls for advancing Community Psychology research and action that would critically interrogate Community Psychology's foundations, methods, and commitments. We were then stirred by the papers then presented at the Eighth ICCP (see Sonn & Fox, 2021), held in Melbourne, Australia, in 2020 that progress these aims. These papers included critical approaches to fostering anticapitalist solidarities, shifting ideologies from inclusion to decolonial solidarities, community psychology and the climate crises, and critical examinations of the role of the academy in producing and reproducing privilege and power. Related to this has been an explicit call to critique the assumptions that underpin hegemonic Western ways of knowing, doing, and being that inform psychology and, specifically, community psychology, and its far‐reaching influence distributed through an uneven global knowledge economy and its circuits of knowledge production and dissemination (Seedat & Suffla, 2017). Inspired by these themes and critical discussions evident at the various International Conferences as well as national ones like SCRA, we have envisioned a special issue that engages with these critical developments from various locations. We focus on solidarities that we understand as relationships rooted in an ethics of care and entail practices that simultaneously unsettle dominant forms of knowledge and embrace ways of knowing and being that advances dignity, community, and nonviolence. In the next section, we describe the various crises that have converged and that many argue have roots in longer histories of colonialism that continues in the present in various forms of structural and symbolic violence, or the coloniality of power and being.

## COVID‐19, COALESCING CRISES, AND COLONIALITY

As we were planning this special issue in 2021, the world was amid a pandemic the impacts of which were bringing into focus the continuing trend of widening social inequalities around the world, revealing entrenched economic, social, and cultural divides (e.g., Perry et al., 2021; Silva & Ribeiro‐Alves, 2021). These divides are expressed in pronounced differences in access, inequity, and inequalities in domains such as health care, food and housing security, safety in public space, dignity and recognition, and educational attainment between those with material and economic privilege and those who are the most vulnerable and under‐resourced in our communities (see Therborn, 2012). The World Health Organisation (2021) and the United Nations Conference on Trade and Development (2021) declared that the COVID‐19 pandemic has done more than show the entrenched nature of unequal wealth distribution and the burden of suffering, it has also highlighted the pandemic as a site of coloniality through practices such as vaccine hoarding, lack of access, pricing, and the uneven distribution of vaccines to poorer nations. To avoid overly deterministic analyses of crises, Hall and Massey (2010) suggest conceiving them as conjunctures, periods in which seemingly contradictory economic, social, political, and ideological elements convene and condense to give societies a specific and distinctive shape.

In many parts of the world before the COVID‐19 pandemic, mass protests were bringing attention to the continuous forms of violence shaping societies. In Chile, for example, feminist‐led protests that had ripple effects around the world called out gender‐based patriarchal violence (Martin & Shaw, 2021). Not long into the pandemic the murder of George Floyd, at the hands of an institution sworn to protect its residents, reverberated around the world igniting uprisings against anti‐Black racism in various cities announcing that Black Lives Matter. In different countries the perniciousness and persistence of state and “private” violence are seen: in the imprisonment of refugees and asylum seekers (Buckingham et al., 2021; Esposito et al., 2019; through the extractivism evident in the blatant disregard of mining industries blasting sacred sites of Aboriginal communities (Muir & Atkinson, 2021); and in responses to the devastating bushfires in Australia, Chile, and the United States that signal intensifying ecological climate crises; rising evidence of “femicide” and violence against women/girls/femmes across the globe (United Nations Office on Drugs and Crime, 2021).

These alarming trends of wilful structural and symbolic violence, of metaphysical catastrophe, many argue, have roots in the long‐connected histories of Empire and colonialism and what decolonial scholars have termed coloniality/modernity (Maldonado Torres, 2016; Quijano, 2000). Mignolo and Walsh (2018) suggested that coloniality is the darker and hidden side of modernity that highlights the deleterious consequences of violence and the enactment of dominant Western ideological assumptions within everyday life that perpetuate systems of power that are harmful to all forms of life. Peruvian scholar Anibal Quijano (2000) coined the term coloniality of power to refer to the continuities of hierarchical relations of exploitation and domination in the present that perpetuate privilege and disadvantage along intersecting structures of sexuality, gender, race, and class.

Maldonado Torres (2007, 2016) and others extended the coloniality of power to include the coloniality of being. Maldonado Torres (2016) draws on the work of Frantz Fanon to theorize the effects of colonialism and coloniality as both systemic and psychological which points to the coloniality of being. Colonization and coloniality have centered on ideologies of race and various mechanisms of control including what wa Thiong'o (1986) refers to as mental control. He notes that: “to control a people's culture is to control their tools of self‐definition in relationship to others” (p. 16). Lugones (2016) extends the notion of coloniality to bring attention to gender and sexual identities within the matrix of power in which binaried and oppositional categories are the basis for social organization placing women in subordinate positions in all domains. Segalo (2020) theorizes the “poison in the marrow,” the residues of colonialism left in our bodies, undigested, and reproduced. These decolonial ideas and concepts have been taken up in community psychology in efforts to create more plural and multi‐stranded forms of activism, scholarships, and critical inquiry.

## PLURIVERSAL AND MULTISTRANDED COMMUNITY PSYCHOLOGIES

With decoloniality at the heart of the special issue, we sought critical projects that were concerned with challenging and disrupting the intersecting dimensions of oppressive power and generating practices that center communality, empowerment, and wellbeing. There was a key desire to read works which expand ecologies of knowledge towards diversality and plurality in ways of knowing, doing, and being. This includes the pursuit of epistemic justice, which involves: “shifting away (delinking) from Western epistemology and engaging nonacademic work where Western epistemology has trickled down framing subjectivities, education, ways of eating, health and destroyed conviviality …” (Mignolo & Walsh, 2018, p. 108). Decoloniality is not about universalizing knowledge; it is concerned with pluralizing and needs to be understood in terms of who is doing it, where, why, and how it is being done. Many authors have begun to explore anew the possibility of decolonial scholarship for community psychologies toward a decolonial standpoint (e.g., Adams et al., 2015; Decolonial Editorial Collective, 2021; Kessi et al., 2022; Malherbe et al., 2021; Reyes Cruz & Sonn, 2011; Sonn & Stevens, 2021). Carolissen and Duckett (2018) provide one example of this study, distilling features of decolonial pedagogy from a collection of 15 papers in a special issue of the American Journal of Community Psychology. They list several markers central to decolonial pedagogy such as drawing on ecologies of knowledge appropriate to the context; disrupting the privileging of Euro‐American/Western epistemologies; reframing pathologized accounts of marginalized peoples; reclaiming and reframing the erasure of histories; deconstructing colonial discourse and inserting counter‐narratives; centering indigeneity and indigenous knowledge in the curriculum, and foregrounding the politics of knowledge production.

Connell et al. (2018) called for greater democratization of knowledge construction and exchange and provided examples of South–North and South–South collaborations between institutions, networks, and alliances that can disrupt the dominance of the center‐periphery dynamic that continue to characterize the global knowledge economy. One aspect of this process is centering and engaging with knowledge formations from majority world contexts, beyond the academy, and in alternative settings. Key examples of critical anticolonial writing from our contexts in Aotearoa/New Zealand and Australia are captured in decolonizing methodologies and Indigenist frameworks articulated by Aboriginal and Maori scholars and activists (e.g., Moreton‐Robinson, 2015; Nakata, 2007; Rigney, 1999; Smith, 2012). The approaches privilege Indigenous and First Nations peoples' realities, honor social mores, and practices on country and lands and understand the context in shaping experiences rooted in cultural values, creation narratives, notions of the interconnected self, and relationality between people and the natural world. These approaches provide an opportunity to revision the field, to critique and rearticulate core commitments and values, such as empowerment, ecology, cultural relativity, and collaboration, and to regenerate situated, deeply theorized, transformative praxis for social change. This praxis entails expanding and building a pluriversal and multistranded Community Psychology that can foster and sustain solidarities for justice, health, and wellbeing in entangled local and global contexts (Dutta, 2016; Langhout, 2016; Serrano‐García, 2020).

In community psychology, we have seen several publications that show efforts to engage with and act toward what decolonial scholars have termed the “decolonial otherwise.” Mignolo and Walsh (2018) define the decolonial otherwise as “the continuous work to plant and grow an otherwise despite and in the borders, margins, and cracks of the modern/colonial/capitalist/heteropatriarchal order. The pedagogies of this praxis are multiple” (p. 101). In a different yet related area of writing on the social movements and their relationship to social transformation and justice projects, Khasnabish (2020; Haiven & Khasnabish, 2014) uses the term “radical imagination” and Maori scholar Graham Smith (2017) used the term “indigenous imagination.” Khasnabish (2020) writes that the term radical imagination refers to our capacity to imagine an otherwise. Haiven and Khasnabish (2014) note that radical imagination is not a thing that people possess; it is collective and processual: “something that groups *do* and do together through shared experiences, shared languages, stories, ideas, arts and theory. Collaborating with those around us we create multiple, overlapping, contradictory and coexistent imaginary landscapes, horizons of common possibility and shared understanding” (para. 4). Decolonial otherwise and radical imagination share commonalities with the critical community and liberation‐oriented psychologies and the various epistemological, methodological, and ethical resources that underpin these fields. In some ways, these resonate with liberation‐oriented psychology's call to: “involve ourselves in a new praxis, and activity of transforming reality that will let us know not only about what is but also what is not, and by which we try to orient ourselves towards what ought to be” (Martín‐Baró et al., 1994, p. 29).

Fernández et al. (2021) map the various trajectories and expressions of decolonial discourse and practice in community psychology and related approaches that are essentially concerned with fostering and enacting solidarity. They note the diverse scholarly roots that include various postcolonial, feminist, and Indigenous authors such as W.E. du Bois, Frantz Fanon, Gloria Anzaldua, Sylvia Wynter, and Linda Tuhiwai Smith. They suggest that the decolonial turn and articulation of a decolonial otherwise is a process that involves ontological and epistemological disruption of hegemonic ways of knowing and doing, that is, naming and contesting the coloniality of power/knowledge in research and practice. As noted by Fernández et al., people enact a new praxis through different yet interrelated orientations, which often involve tensions and challenges as they navigate ethics and power in charting new routes toward a decolonial otherwise. Central to the pursuit of the decolonial otherwise is the notion of solidarity and the tensions and challenges that emerge as differently positioned social actors come together to tackle oppression and promote liberation.

## FOSTERING SOLIDARITIES AS CRITICAL RELATIONAL PRAXIS

The current special issue continues and expands on these engagements with decolonial discourse and practice in community psychology by exploring solidarities. The notion of solidarity is central to relational practice and dialogue. The Maori term for solidarity is “kōtahitanga,” which means to work together for a common purpose. In Western knowledge traditions solidarity as a term has deep roots in sociology with reference often made to the concepts of organic and mechanical solidarity introduced by Emile Durkheim (Kivisto, 2017). Mechanical solidarity refers to coming together around shared values and organic solidarity is the product of interdependencies produced by segmented social arrangements. Other sociologists have discussed solidarity as a union between the one and the many where diverse actors can come together and stand with each other. Hunt and Benford (2004) wrote that solidarity is rooted in networks of relationships linking people—bodies of people and spirit that involve feelings of identification. They note that “solidarity is an identification with a collectivity such that an individual feels as if a common cause and fate are shared” (p. 439). Along similar lines, Coates (2007) wrote that “Solidarity, defined as the perceived or realized organization of individuals for group survival, interests, or purposes, may result from either external threats or internal needs” (p. 4620). Others have pointed to the various levels at which people have explored solidarity such as in terms of identification, space, movements, organizations, and transversally, as people navigate, contest, and generate possibilities for acting together on the world to effect change.

Solidarity has arguably always been central to the goals of community psychology and has been expressed as a commitment to work in collaboration with differently positioned social actors towards social justice and systems change (Nelson et al., 2001; Rappaport, 1977). There is a commitment to social justice, relational ethics, and critical reflexivity that is foundational to creating praxis across and within border spaces alongside marginalized groups (Hodgetts et al., 2021). This is resonant of liberation psychology's “preferential option for the poor,” often understood as the marginalized, oppressed, and excluded in various contexts within systems and practices of domination (Burton & Kagan, 2005; Freire, 1972; Montero et al., 2017). Central to this effort is situated knowing, relationality and a dialogic orientation, that is, solidarity as the process. Such an orientation:

is expressed in the need to incorporate the cultural knowledge and the people's voices. The need to understand everything happens in social relationships, and that the other in those relations has to be not only acknowledged, but also heard and answered (Montero & Sonn, 2009, p. 2).

Writing in different disciplines that theorize solidarity is relevant to our interest of how people foster and sustain solidarity or practices for solidarity in research and action (see Land, 2015). Solidarity, as a relational practice, as noted by Jennings (2018), “inherently leads us to view our own lives and agency as bound together with the rights, well‐being, health and dignity of others here and now” (p. 557).

Jennings (2018) offers a way of understanding standpoints of solidarity that is tied to ethics of care and regards the “gesture and stance of solidarity” (p. 557) as an important public signal of recognition for the moral standing of a person or community. They suggest solidarity relationships can be framed as postures that one assumes toward others whose moral standing requires bolstering, and they are standing up for, standing up with, and standing up as. Each of these postures denotes a different standpoint in relation to self and other and deeper ethics, recognition, and relationality.

Standing up for, takes an advocacy stance against oppression and exclusion and involves practices such as assistance, defending, and pleading for the other. Within this, the other also can refer to

other species, an ecosystem, or a cultural way of life. What is crucial is that there is some kind of power or knowledge differential between self and other in a relationship of solidarity and some kind of injustice or danger impinging upon the life of the other (p. 557)

Jennings notes that this position does not necessarily challenge the underlying basis for social inequality and that if the structural elements are not addressed “standing up for can perpetuate subordination rather than achieve equality” (p. 557). Standing up with, goes further in the direction of a “recognition of moral standing. Moving from standing up for, to standing up with, requires deeper engagement with the experience and lifeworld of the other” (p. 557–558). Standing up necessitates the capacity to embrace ontological possibilities and lifeworlds other than or outside one's own. This expansion of possibilities is more likely to engender respect, rather than mere tolerance, and fosters more humanizing and reciprocally beneficial relationships.

The third orientation, standing up as, suggests a:

stronger degree of identification between the providers of solidaristic support and the recipients of such support. The solidarity of standing up as, involves finding a kind of covering connection that does not negate diversity among individuals at all, but rather establishes the grounds of its respect, protection, and perpetuation (p. 558).

Each of these orientations entails differential social standing and positioning in and across contexts, communities, and institutions, and entail negotiating attendant dynamics of power. There are numerous examples of efforts to strengthen community praxis (i.e., the interconnected cycles of reflection, research, and action) within various contexts characterized by dynamics of oppression produced by hierarchized social systems in local and global contexts.

For example (Torre et al., 2017), advance the critical praxis of participatory action research (PAR) as it opens the possibility of building solidarities rooted in inquiry and in struggles entrenched in and across fault lines of privilege and dispossession. Drawing on occupations and state violence in both Palestine and the Bronx New York, Fine (2015) sketch a framework for inquiry/praxis in which we are:

Searching these days for binary busters … solidarity seekers; those who stand for, against and with; those who are firmly grounded in critical analyses of community but/and/also/therefore carving common ground with Others across dangerous power lines. These people make, and remake, communities in and across place, and they challenge our academic understandings of how communities and cultures emerge, sustain, transform and remix (p. 6).

Ellison and Langhout (2020) examine how community organizers address the reproduction of such systems of oppression. The authors suggest that intersectional solidarity can be understood within a framework that considers relational–corporeal praxis. They note that:

relational labor and corporeal literacy practices are overlapping, as both require relational and internal processes. Relational labor, including social and emotional support, is work that individuals do for others. Corporeal literacy practices (i.e., recognizing and attending to information created within one's body) occur in relationship with others in the social world …, and is a kind of work individuals must do so they can work in solidarity … (Ellison & Langhout, 2020, p. 965).

Smolović Jones et al. (2021), in a study of a women's rights protest movement in Montenegro, present an account of feminist solidarity as an embodied practice that involves contestation and difference experienced through the body. These experiences are generative providing insights into solidarity as “a participative and inclusive endeavour driven by conflictual encounters, constituted through the bodies, language and visual imagery of assembling and articulating subjects” (p. 917). The authors illustrate solidarity practice as agonistic, as emerging from and fostered in contexts of contestation and generativity.

Tuck and Yang (2012) and Gaztambide‐Fernández (2012) caution against an uncritical embrace of solidarity that risks reinscribing colonial logics and moves toward white innocence in settler colonial contexts. This has been echoed by Land (2015) and Sonn and Quayle (2013; Quayle & Sonn, 2013) who explored the barriers and opportunities to build partnerships between Indigenous and nonindigenous settlers in Australia. Walking with Indigenous people in south‐east Australia, Land (2015) explored “sticking points” in the ways nonindigenous peoples engage with efforts of solidarity and activism. Land highlighted some key principles for solidarity, including decentring White people, supporting First Nations‐led initiatives, understanding and positioning within culture and history, and engaging with “humility” and “letting go of knowing” (Land, 2011, p. 60). Solidarity requires that we recognize our roles, responsibilities, service, and humility, as well as our positionalities in contexts, knowing when supporting from a distance and stepping back is required. Refusing a neo‐liberal construction of justice as zero‐sum, but as profoundly interdependent, solidarity work requires dialogic labor to uncover spaces of the common good and to address, with intention, sites of difference/power to work through.

Many writers (see Harrel & Bond, 2006) emphasize the complex, entangled, the messiness of researching in the spaces and places where people differently positioned contest meanings, ideologies, and power relations. These settings or encounter spaces are important for exploring, reflecting, and reporting on the tensions, contradictions, failures, limits, and boundaries that systems create in many radical practices. Exploring and reflecting on such tensions can offer means to further resist or contest them. Reporting on such tensions is a way to promote transparency in radical work and can be positioned in itself as a decolonial practice. Such presenting of failure, contradiction, tension, of multiple realities where success, failure, and experiences otherwise can coexist, is discouraged in so‐called scientific and academic spaces where universal truths, objective realities, and perfect practices are encouraged thereby producing wilful ignorance of the limitations and violence of this way of knowing and doing. It is within the context of this broader scholarship that we sought to engage with colleagues in dialogue about solidarity praxis in community research and action.

## FOSTERING AND SUSTAINING SOLIDARITIES AS A TRANSNATIONAL CRITICAL COMMUNITY PROJECT

We received a broad range of papers in response to the call. Two of the papers received were written by authors who came together at the online conference held in Melbourne (Ciofalo, 2021; Fernández et al., 2021). While the papers are diverse in terms of issues, approaches, populations, and methodologies, we were able to distill several themes that respond to the questions that we set out to explore. It is clear that they are building from solid foundations of solidarity‐oriented work evident in ways of knowing, doing and being that have been made invisible or are often omitted. In this collection, we see efforts to construct community psychologies otherwise, with criticality, humility, and a sense of urgency to take seriously the obligation of the academy to honor and support struggles on the ground.

Most of the writing in this special issue reflects on *process* rather than results or solutions. Critical discussion of epistemic positions, values employed in work, tensions experienced, and on the relationality of work can be hugely valuable to those reading—especially those younger scholars and students, community activists, and policymakers eager to understand how to engage “with” and “for” rather than “on” or “about” communities in crisis. While situatedness and specificity should never be eroded, they offer opportunities for application to other spaces and peoples that results of applied research do not necessarily offer; a way in which global solidarities can be performed. Fine et al. (2007) has called this “provocative generalizability”—the capacity for a piece of research conducted in one community to incite/spark resonance in a site far away geographically and yet intimately familiar in terms of oppression/resistance. That is, we are committed to understanding community psychology as a praxis within localities and also to make visible how circuits of oppression, privilege, and resistance travel across; both crucial to imagining how things might be otherwise.

The papers featured in this special issue respond in several ways to the call with a focus on processes and praxis. The authors write about forms and circuits of violence and efforts of solidarity between academics, communities, and other actors. We offer our reflections on what we have learnt from reading the rich and diverse contributions to this issue, all in one way or another, concerned with fostering solidarities through the critical task of exposing and transforming coloniality and its expressions in varied scales, structures, and intersecting forms of violence such as racism, sexism, femicide, ableism, deportation, and extractivism. Our desire as co‐editors was not to enclose the plurality of solidarities expressed in the papers in an unyielding methodological or conceptual framework, but rather to hold them lightly within thematic spaces as invitations for readers to consider. Across the papers authors mobilize border knowledge and thinking, knowledge that have been neglected, omitted, or relegated below the abyssal line, the line used describe economic, social, cultural, and linguistic divides between those in the Global North and Global South (Santos, 2016). Importantly, the authors show how they enact epistemic disobedience and the “creative joy of knowing beyond the disciplines” (Mignolo & Walsh, 2018, p. 225), which is central to pursuing the decolonial option. We now turn to the papers in this special issue that we have organized around three thematic spaces: Ecologies of being and knowledge: Indigenous knowledge, Networks, and Plurilogues; Naming coloniality in context: Histories in the present and a wide lens; and Relational knowledge practices: Knowing beyond disciplines.

## ECOLOGIES OF BEING AND KNOWLEDGE: INDIGENOUS KNOWLEDGE, NETWORKS, AND PLURILOGUES

A key feature of the decolonial option is to unlink from the Western hegemonic modern forms of knowing and doing and to embrace indigenous knowledge systems and ways of knowing, doing, and being that have been excluded or silenced (Dudgeon et al., 2020; Suffla & Seedat, 2021). In this issue, there are several examples of knowledge from the margins/below the abyssal line such as—indigenous, feminist, victims of violence, and asylum seekers' voices. Ciofalo et al. (2022) uses the concept of coloniality of power/knowledge to unsettle the dominant narrative of community psychology in the North. Quoting Serrano García (2020), the author suggests that community psychology in the US has been ethnocentric despite its expressed commitment to cultural relativity and context. Serrano‐García (2020) notes that:

I am continuously surprised when I attend different events in the U.S. and hear colleagues identifying gaps in the field which have already been attended to in other countries. For example, Latin America was way ahead of the U.S. in qualitative and participatory research, Freirian models, and community interventions. To fill similar gaps, colleagues must travel and learn other languages so that they may collaborate in projects and access literature published in other tongues (p. 14).

Ciofalo et al. (2022) reviews community social psychology from Abya Ayala, the name of the American continent given by the Kuna Indigenous peoples from Panama. They argue that decolonial paradigms based in the epistemologies of the Global South offer another path to delink “from Western‐centric ideologies that are not anthropocentric and promote sustainability, epistemic and ecological justice, and *Sumak Kawsay/Buen Vivir* (wellbeing) that includes the rights of the Earth”(p. 289).

In their transnational dialogue, Ciofalo et al. (2022) provide examples of indigenous psychologies from Mexico, Australia, and Aotearoa/New Zealand. The authors state that “Indigenous psychologists propose that their own religions, origin narratives, and philosophies, their own epistemologies, praxeologies, and axiologies, form the fertile ground upon which to develop particular psychologies as well as research methodologies” (p. 431). in one example, they describe the Aboriginal and Torres Strait Islander Social Emotional Wellbeing model that centers indigenous knowledge, cultural resilience, connection to Country, and self‐determination. A second example shows the development of *Māori Psychologies* within a broader agenda that centers on Māori autonomy and self‐determination. A third example describes Mayan Indigenous Psychologies.

Another important contribution by the different papers is the linking across contexts in practice and by adopting theoretical approaches that are attuned to global entanglement, beyond the local nation‐state, and that show various circuits of dispossession and privilege (Fine & Ruglis, 2009) across communities, without flattening how fundamentally difference/place/power matter. Hagelskamp et al. (2022), Marinkovic et al. (2022), and Suarez‐Balcazar et al. (2022) provide examples of transnational participatory and collaborative projects that seek to promote social justice and change that recognizes local and global relationships, the geopolitics of knowledge, and the coloniality of neoliberalism and extractivist economies. These are examples of research solidarities of standing up and with—across national borders. Hagelskamp et al. reflect on People Powered, a transnational network of civil society organizations that challenge hierarchies, supporting poor communities to take part in participatory democracy, based on participatory budgeting—participating in local decisions being made by governments and institutions. Through their analysis of organizational documents, interviews with people from the network in Africa, Asia, Eastern, and Western Europe, and reflections they show how People Powered enact decoloniality in everyday practices. In People Powered decoloniality is enacted through situating actions in the histories, epistemes, and values of places, and also engaging internationally in creating space for communities to engage in dialogue and solidarity. The work shows actions toward disrupting hierarchies powered by neoliberal policies and globalized capitalist exploitation in a local context aimed to “help everyday and especially historically marginalized citizens to be taken seriously by authorities” (Hagelskamp et al., 2022, p. 295).

Marinkovic et al.'s (2022) work also show the power of transnational networks and collaboration. In their projects, adult academic researchers and child coresearchers engaged in transnational dialogue with the aim of fostering solidarities between them to progress participatory research with children, who are historically marginalized from decisions made about issues that affect them. Their work describes ways in which they: “share resources, build capacities, and cocreate knowledge that incorporates the perspectives of people from diverse contexts and disciplines” (p. 308). They suggest that “the goal of scaling‐up participatory research with children nationally and internationally to gain a better understanding of children's perspectives on different issues affecting their wellbeing and the pathways for action they propose to address them” (p. 308). Suarez‐Balcazar et al. (2022) offers a different approach to fostering dialogue about community‐based participatory research in three countries: the United States, Spain, and Peru. Each of these studies shares a commitment to accompany groups in the pursuit of justice, which is often “related to efforts to attain personal as well as collective well‐being” (p. 318). These authors document the combination of factors that produce inequity in employment for people with disabilities in the United States, and a participatory program developed with people to provide opportunities and resources to support their becoming self‐employed. The study from Spain documents the differential economic impact of the COVID‐19 pandemic on Roma women and girls in already marginalized neighborhoods. They describe a participatory program of community advocacy designed to ensure city officials heard the voices of these groups in the distribution of resources to ameliorate the impact of COVID‐19. The study from Peru documents the violence and terror that lead to the displacement of women and their search for fairness and dignity in and away from home. They describe participatory efforts to support the women and their desire to return home. Through their dialogue, these authors document injustice and forms of violence and call for:

redefining the concept of social justice and addressing the failure to achieve it considering the current problems in which we are living and our inability to prevent as well as to rectify inequalities and ensure a fair redistribution of natural, technological, and social resources (p. 319)

Some papers in the issue offer lessons from the processes of collaborating across and from different geopolitical locations of enunciation including Ireland (Vine & Greenwood, 2021); Hawaii (Sasa & Yellowhorse, 2022); Northern India (Dutta et al., 2021) around topics that may seem separate but are entwined at the root: issues of displacement, structural violence, youth development, poverty, ableism, and racism.

Instead of typical comparisons of strengths and weaknesses, these projects adopt a methodology of diffraction (Langhout, 2016) to theorize practices, actions, and effects in and from the place. The authors make visible the importance of location and epistemes and the flows of knowledge, worldviews, and the dynamics of the knowledge economy. Importantly, as a result of the transnational approaches adopted and the emphasis placed on situated knowing and relationality, these authors have offered examples of how methodological nationalism (Wimmer & Schiller, 2003) can be contested and pathways for democratized transnational knowledge exchange opened (Connell, 2019).

## NAMING COLONIALITY IN CONTEXT: HISTORIES IN THE PRESENT AND A WIDE LENS

The various papers in the issue focus on different domains of oppression and spheres of coloniality that are rooted in the colonial matrix of power, capitalism, and rampant inequities and inequalities in access to resources. Ummel et al. (2021), make a case for pandemic grief that has resulted because of COVID‐19‐related suffering. As many are doing so in response to the pandemic, they suggest that in societies organized around capitalist ideologies and structures, risks and hazards are unevenly distributed, with poor and racialized communities carrying the burden of suffering. Vine and Greenwood (2021) and Saleem and Li (2022), in Ireland and the United States, respectively, highlight the challenges faced by displaced people, including migrants, asylum seekers, and refugees who move to seek out safety, security, and protection in a different country. Displaced people are not always received with open arms and have to negotiate power expressed in negative discourses and social representations rooted in ideologies and structures of White supremacy, deficit views, and racial privilege that dehumanize different groups and attendant racism and sexism expressed in everyday and institutional contexts in receiving communities. Vine and Greenwood write about the efforts of allies and collaborators in the receiving communities who stand up and with, as a form of political solidarity, with displaced groups by providing social support, advocacy, and support for the creation of settings, such as community solidarity initiatives, from which to build connections and to advocate on behalf of those excluded. Saleem and Li argue that colonial racialized logic continues to shape migration policies in the United States. They highlighted the problematic language that constructs displaced people as illegal and refer to this as a form of border imperialism. The authors argue that a wide lens is required to understand oppression which includes:

…the historical and ongoing *erasure* of the Indigenous people, the *enslavement* and *incarceration* of black people, and xenophobic and nationalist *exclusion* and *deportation* of “illegal aliens” that include largely migrants from the global south (e.g., central and South America, the African and Asian continent) … (p. 382).

In addition, Sasa and Yellow Horse (2022) show how census methodology as an epistemic practice works as a mechanism of coloniality through race/ethnic‐based classification systems, in particular, the effects of misclassification and conflation within the United States of “Native Hawaiians and Pacific Islander” as “Asian or Pacific Islander” which fails to recognize the diversity and complexity of ancestry of Native Hawaiians. Sasa and Yellow Horse consider the “invisibilising” of Native Hawaiians through pan‐racial categorization approaches as a form of “invisibilising” the role of US colonialism and erasing the injustices experienced by Native Hawaiians using an Indigenous lens, as well as the marginalizing experiences of Pacific Islanders more broadly. Sasa and Yellow Horse provide this as another example that calls for decolonial practice through indigenizing research and practice.

Using letter writing, Fernández et al. (2021) creatively and powerfully stitch together a plurilogue through letter‐writing from and across places. One of the aspects made visible through the letters is the deep roots of sexism and other forms of violence in colonial and patriarchal systems and their expression in specific places, in Indonesia, Mexico, the United States, and India. Dutta et al. (2021), through innovative decolonial accompaniment, show how the Miya in Northeast India build communities of resistance against state‐sponsored structural and cultural violence rooted in colonial histories. Escobar (2021) names state violence in Colombia and its effects and long‐lasting consequences for survivors. They focus on the struggles of survivors and their practices of remembering and healing in pursuit of justice. The different papers name the accumulation of dispossession, the violence of erasure, colonial narcissism, and the effects of individualism in and outside the academy. The articles also bring to the forefront theorizing and analysis that takes a long and wide view of the interconnected systems of violence and how these continue in the present, and also how local dynamics of oppression are expressed at global levels. They do not only point to the circuits of violence. Indeed, where there is violence there is also resistance, struggle, and circuits of solidarity. Importantly, across all the papers the authors trouble the binaries of university‐community, calling for new roles, practices, and ways of knowing, being, and doing: Dutta et al. expressed it in this way: “Through a politics of location and engagement, we actively negotiate our varied identities and complex relationships to hegemonic power and write from our relationally rooted places of love, care, and accountability” (p. 357).

## RELATIONAL KNOWLEDGE PRACTICES: KNOWING BEYOND DISCIPLINES

Relational ontology, epistemology, and ethics are key coordinates for critical community research and action. These practices are encapsulated in the powerful expression “Nothing about us without Us.” Relational knowledge practices depart from an “…ethic of connection, of mutually implicated humans whose primary duty is to respond to the calls of others, particularly those who are vulnerable …” (Rose, 1994, p. 20). This position has been echoed by various standpoint approaches that contest zero‐point epistemologies of neutrality in favor of a clear locus of enunciation. For example, from a feminist perspective, relationality, ethics, and transversality are made central and inquiry is relocated on the ground where:

knowledge is made, negotiated, circulated; and where the nature and conditions of the particular ‘ground’, the situations and circumstances of specific knowers, their interdependence and their negotiations, have claims to critical epistemic scrutiny equivalent to those of allegedly isolated, discrete propositional knowledge claims. (Code, 2006, p. 4)

Indigenous perspectives go further to emphasize the recovery of cultural memory, ethics, relational conceptions of self, culture, and the natural world. In these articles, we see examples of epistemic disobedience and the “creative joy of knowing beyond the disciplines” (Mignolo & Walsh, 2018, p. 225). Authors in this issue advance approaches and methods that elevate “the lived concerns and pedagogical imperatives of freedom, anguish, responsibility, embodied agency, sociality and liberation…” (Mignolo & Walsh, 2018, p. 2). This resonates with Fine's (2018) call for just research that embraces a wider methodological imagination to effect social change and performative social science that are inclusive, polyvocal, and democratic (Gergen & Gergen, 2010), but also ways of knowing and doing advocated by Indigenous scholars and activists, critical race scholars, feminist theorists and other approaches that have been marginalized in whitestream approaches to knowledge projects. Fine (2021) notes that “These activist scholars and texts have suffered “death by canon,” whited‐out from the imperial, positivist, and universal‐law seeking/power flattening history of psychology, through acts of Epistemicide” (p. 61).

The knowledge practices in the issue include autoethnography and collective autoethnography (Drake et al., 2022), storytelling and counterstorytelling (Dutta et al., 2021; Escobar, 2021) letter writing (Fernández et al., 2021), photo‐elicitation (Vine & Greenwood, 2021), co‐interviews (Saleem & Li, 2022; Vine & Greenwood, 2021), poetry and other forms of expression (Dutta et al., 2021). Fernández et al. (2021) in using letter writing, a method with a long history in feminist approaches write that:

Letters transcend and trespass; they also thread. They are the needle that weaves, el hilo y la aguja, our stories alongside the longings for connection, relationality and radical solidarities that are grounded in what is felt in body, bone and flesh, in the marrow of our soul. Letters connect and amend when words are lost, when we cannot express verbally or even physically what is felt (p. 401)

The authors emphasize the role of letter writing as a means for deconstruction, dialogue, and accompaniment. For these authors letter writing as a decolonial feminist practice provided means to “unravel the yarns of the academy that entangle us, not alone or isolated but rather in the company/accompaniment of each other; we have no other ways to exist and resist the academy” (p. 401). Dutta et al. (2021) along similar lines engage in a methodology of refusal that “… requires a departure from theory/research that render Miya people/issues/struggles as inherently knowable, as objects of study.” For this collective also draws from feminist traditions of theorizing from the flesh to “… generate theory *from* embodied, lived experiences, not *about* it.” These papers also highlight the central role of arts and aesthetics as ways of knowing and doing and as sites of resistance and modalities for reparation, restoration, and healing. Anzaldúa and Keating (2015) wrote about the transformative role of arts and creativity; they named it as a liberatory impulse stating: “when art functions as a spiritual discipline, the work of mind and body come together in acts of imagination” (p. 40–41). Importantly we also recognize that the written word has a history interconnected with colonizing groups constructing “others,” doing so on and without “body of knowledge, both historical and ongoing, that is produced by others “about us,” across a range of intellectual, government and other historical texts” (Nakata, 2007, p. 7).

Drake et al. (2022) use collaborative autoethnography to explore how “as ‘Westerners’” they “might confront what is known as “Western’ psychology.” They engage in critical practices, decolonizing the mind, in contact zones, creating a third space from which to enact radical imagination, in line with what Graham Smith (2017) calls for the enactment of an “indigenous imagination” often suppressed by colonialism. Through this embodied approach they opened up a space “to consider the ways we collectively have been implicated in wider coloniality.” Escobar (2021) accompanies survivors of state violence guided by ethnography to show how “embodied memory becomes a practice of resiliency that transmits purpose to the lives of survivors in the present and how this relationship with the past and ongoing violence shapes the justice centered actions and visions for the future.” In another example of responding to the pain, described as pandemic grief resulting from COVID‐19 pandemic‐related suffering, Ummel et al. (2021) describe a solidarity‐driven response based on the notion of compassionate communities. The authors describe codesigned activities that were developed to stand with people through an online community. They note that “Individuals naturally have the impetus to express solidarity and come together to compassionately support each other and can do so in a way that also tackles wider social injustices, an issue that professionalized, privatized help cannot solve” (p. 378).

The relational practices enacted in this issue echo Martín‐Baró et al. (1994) call for responsive and accountable psychology practices in the notion of accompaniment:

the choice is between accompanying or not accompanying the oppressed majorities…. This is not a question of whether to abandon psychology; it is a question of whether psychological knowledge will be placed in the service of constructing a society where the welfare of the few is not built on the wretchedness of the many, where the fulfillment of some does not require that others be deprived, where the interests of the minority do not demand the dehumanization of all (p. 46).

There is growing attention given to knowledge emerging from various borderlands ‐ university‐community, race/class/gender/sexuality; research‐action, and ways of doing and being. In these spaces, people are working in solidarity in pursuit of equity, recognition, and epistemic justice.

## CONCLUSION

There are many ways that we could have organized this issue, but we have, through editorial collaboration, arrived at the following thematic spaces: (1) ecologies of being and knowledge: Indigenous knowledge, networks, and plurilogues; 2) naming coloniality in context: histories in the present and a wide lens; (3) relational knowledge practices: knowing beyond disciplines. The authors of the papers highlight the tensions and challenges that they navigate in efforts to accompany differently positioned people in their struggles on the ground, coming up against borders and coloniality deeply rooted in the matrix of power, neoliberal systems, globalized capitalism, and structures underpinning ideologies of supremacy, meritocracy, and individualism that shape and constrain people's capacities to live dignified lives. Collectively, in and across places, researchers wrestle with expressions of coloniality in research and practice, with enacting relational ethics, situated knowing, and a decolonial attitude in the discipline, in unsettled times, and through solidarity praxis.

There is a renewed sense of urgency to foster community‐oriented psychologies that cultivate relationships and partnerships with various communities in their struggles against coloniality and oppression (Maldonado Torres, 2016). Rua et al. (2021) noted that we need to “…respond and prioritise and address the intergenerational grief and pain endured by our people” (p. 179). The call for various social actors to join locally and across with “like‐minded scholarly activists who share our burning sense of urgency in addressing the needs of our communities and for decolonising psychology and society‐at‐large” (p. 179). The unsettling of epistemic institutions and their histories of marginalization, oppression, and violence is part of this ongoing call made by scholars around the world and by Indigenous scholars in Australia for whom the quasi‐neutrality of dominant ways of knowing is complicit in the project of coloniality. Watkins (2021) commented that accompaniment, as an approach, requires a fundamental reorientation for those who have been born into and/or educated into white, economic, and/or social privilege. This vantage point brings into focus privilege/power, as noted by Fernández et al. (2021). Chelsea Watego (2021) stated:

… to take up the position of proclaimed objectivity is tantamount to taking a side, the wrong side. Those supposed neutral and impartial processes always seem to stack the odds against Black people, and their conclusions are always in solidarity with the perpetrators, and in harmony with the innocence they see in themselves. The idea that there is an objective take is one grounded in white universalism and supremacy, of seeing racism as occasional and aberrational, if one sees it at all (para. 31).

This includes, but expands on, the feminist ideas of strong objectivity and positionality (Harding, 1992), to encompass a “decolonial attitude” rooted in Fanon's theorizing (Maldonado‐Torres, 2017). The decolonial attitude centers humanization and demands a rethinking of the:

complicity as psychologists in global and local injustice” but does so with a call for those working from positions of relative privilege to “get up from their desk and join the protest, not as leaders but in solidarity with community organizations. (Decolonial Psychology Editorial Collective, 2021, p. 8)

Unsettling epistemic power held by disciplines, universities, and policymakers can be progressed through building solidarities, locally and transnationally, and repowering community narratives that are rooted in collective self‐determination, truth‐telling, healing, the recovery of historical memory, and the cocreation of settings from and with, to stand up for, as and with. Truth‐telling locates historical memory and decolonial praxes within solidarities, but also engenders trust as the way in which solidarities can enact change. Arts and aesthetics can play a central role in provoking the otherwise, to act against numbing forms of structural and symbolic violence. In this issue, we sought to drive the conversation from “peripheries” back to the “center,” with examples of critical inquiry and emancipatory practices and orientations toward solidarities in and across contexts. We build on the efforts of others who have gone before and whose work are not in canons but who have carved pathways in the hope that we ignite solidarities across and in the field. We invite you to engage with us and the various contributors to this special issue in our efforts to foster and sustain transnational solidarities for decolonial community research and action.
